# The outcome of primary brachial plexus reconstruction in extended Erb’s obstetric palsy when only one root is available for intraplexus neurotization

**DOI:** 10.1007/s00238-017-1302-2

**Published:** 2017-03-30

**Authors:** Mohammad M. Al-Qattan, Amel Ahmed F. El-Sayed

**Affiliations:** 1 Department of Surgery, King Saud University, PO Box 18097, Riyadh, 11415 Saudi Arabia; 2 Department of Obstetrics and Gynecology, King Saud University, Riyadh, Saudi Arabia

**Keywords:** Obstetric brachial plexus, Nerve transfer, Nerve graft

## Abstract

**Background:**

A recent review by the International Federation of Societies for Surgery of the Hand showed no studies comparing the results of nerve grafting to distal nerve transfer for primary reconstruction of the brachial plexus in infants with obstetric brachial plexus palsy (OBBP). The aim of this retrospective study is to compare two surgical reconstructive strategies in primary reconstruction of the brachial plexus in extended Erb’s obstetric palsy with double root avulsion: one with and one without distal nerve transfer for elbow flexion.

**Methods:**

Two groups of infants with extended Erb’s palsy and double root avulsion were included in the study. Group I (*n* = 29) underwent reconstruction of the brachial plexus without distal nerve transfer. In group II (*n* = 26), the reconstruction included a distal nerve transfer for elbow flexion.

**Results:**

Both groups had an excellent (over 96%) satisfactory outcome for elbow flexion. Group II has a significantly better outcome (*P* < 0.05) of shoulder abduction and wrist extension than group I.

**Conclusions:**

The use of a distant nerve transfer for bicep reconstruction in extended Erb’s obstetric palsy with double root avulsion gives a better outcome for shoulder abduction and wrist extension; and this seems to be related to the availability of more cable grafts to reconstruct the posterior division of the upper trunk and the middle trunk.

Level of Evidence: Level III, therapeutic study

## Introduction

There are several large series describing the results of primary exploration and reconstruction of the brachial plexus in infants with obstetric brachial plexus palsy (OBBP) [1–9]. A recent review by the International Federation of Societies for Surgery of the Hand showed no studies comparing the results of nerve grafting to distal nerve transfer for primary reconstruction of the brachial plexus in infants with OBBP [10]. The committee concluded that surgeons need to avoid an over-reliance on nerve transfers and should be more inclined for nerve graft reconstruction [10].

In extended Erb’s palsy (involving the C5, C6, and C7 roots) with double root avulsion, only one root is available for intraplexus neurotization. Distal nerve transfers for biceps reconstruction in OBBP were first introduced by Al-Qattan in 2002 who described the use of Oberlin nerve transfer (ulnar nerve fascicle transfer to the biceps nerve) in two cases of OBBP [11]. Our obstetric brachial plexus clinic was established in 1995, and we started using distal nerve transfers for elbow flexion in primary reconstruction of OBBP in 2002. This gave us the opportunity to compare two primary surgical reconstructive strategies in infants with extended Erb’s palsy and double root avulsion: reconstruction of elbow flexion by intraplexus neurotization in one group and by distal nerve transfer in the other group. This paper is a retrospective comparative study of these two groups of patients.

## Patients and methods

Ethical approval was obtained for this retrospective study. The study included all infants (seen between 1995 and 2013) with extended Erb’s obstetric palsy and double root avulsion; and who were treated surgically by primary reconstruction of the brachial plexus before 6 months of age. Patients who were operated upon after 6 months of age and those who had a follow-up of less than 3 years were not included. It should be noted that the definition of root avulsion in the current paper is “complete” avulsion of the root; and this was confirmed by the presence of an “empty” foramen as well as the presence of a visible bulge of the dorsal root ganglion in the avulsed root. Infants with partial avulsion (a lesion classified by Terzis [1] as avulsion-rupture injury) and infants with avulsion in situ (defined by Al-Qattan [12] as a pale root which is still attached to the foramen, but with zero response to electric stimulation) were not included in the study.

The study population was divided into two groups. In group I, primary reconstruction was done by spinal accessory to suprascapular nerve transfer and by intraplexus neurotization of the upper and middle trunks using the available (one) root of the brachial plexus. In group II, primary reconstruction was done by spinal accessory to suprascapular nerve transfer, transfer of a fascicle from either the ulnar or median nerve to the biceps nerve, and intraplexus neurotization of the posterior division of the upper and middle trunks using the available (one) root of the brachial plexus.

In our center, routine motor assessment of relevant functions is done according to the scores shown in Table 1; and the table also defines what is considered as a “satisfactory” functional outcome. We generally do not offer secondary procedures (such as tendon transfers, osteotomies) for patients who reach these satisfactory scores.

**Table 1:** Postoperative motor assessment in children who underwent primary brachial plexus reconstruction

Function	Scoring or measurement of function	Definition of a satisfactory functional outcome
Shoulder abduction	Measured as degrees of shoulder abduction	Abduction 120° or more
Shoulder external rotation	1 = The hand reaches the abdomen or thorax; 2 = The hand reaches the mouth; 3 = The hand reaches the ear; 4 = The hand reaches the occiput; 5 = normal external rotation	A score of 3 or more
Elbow flexion and extension	0 = no motion, 1 = active motion with gravity eliminated, 2 = active motion against gravity, 3 = active motion against resistance reaching ≤½ normal range, 4 = active motion against resistance reaching >1/2 normal range, 5 = normal	A score of 4 or 5
Forearm pronation and supination	1 = over-pronated forearm causing a functional or cosmetic disability 2 = over-supinated forearm causing a functional or cosmetic disability 3 = functional forearm position (mid pronation—supination or slight pronation) with no or minimal active motion 4 = same as 3 but with good (over 20°) active pronation/supination 5 = normal range of motion	A score of 3 or more
Wrist extension	0 = non-functional (no active extension or extension only with gravity eliminated) 1 = active wrist extension against gravity to less than neutral 2 = active wrist extension against gravity to neutral or more than neutral 3 = normal wrist extension	A score of 2 or 3
Digital extension	0 = non-functional (no active extension or extension only with gravity eliminated) 1 = active digital extension against gravity to less than 1/2 range of motion 2 = active digital extension against gravity to more than1/2 range of motion 3 = normal digital extension	A score of 2 or 3

Note that wrist flexion and hand function are not included in the assessment because C8-T1 roots are not affected in extended Erb’s palsy

Our patient population included many referrals from the entire country. Compliance with postoperative physiotherapy is usually much better during the first 3 years after surgery. Later on, many cases stop physiotherapy; inviting the development of secondary contractures. Hence, we felt that assessment of a satisfactory outcome at the final follow-up in the current comparative study is not appropriate because group I was done before 2002 and group II after 2002. Hence, group I tended to have longer follow-up time and this would bias the results. Therefore, we decided to document the results in both groups at the 3-year follow-up point only; which is also the minimum follow-up required to be included in the study.

The following data were collected at 3 years postoperatively: the number of patients in each group, timing of surgery, intraoperative findings, method of primary reconstruction of the brachial plexus, motor assessment, and percentage of a satisfactory outcome at each relevant motor function. The percentage of satisfactory outcomes was compared between the two groups using the Chi-Square or Fisher’s exact tests. A *P* value less than 0.05 was considered significant.

## Results

Group I included 29 patients with unilateral extended Erb’s palsy and double root avulsion; and the timing of surgery ranged from 4 to 6 months (mean of 5.2 months). Group II included 26 patients with unilateral extended Erb’s palsy and double root avulsion; and the timing of surgery ranged from 4 to 6 months (mean 5.3 months). Intraoperative findings were similar in both groups with the majority showing C5 root rupture and avulsion of the C6 and 7 roots (Table 2). The method of reconstruction is shown in Table 3. In both groups, shoulder external rotation was reconstructed by spinal accessory to suprascapular nerve transfer. The main difference between the two groups is the method of elbow flexion reconstruction. In group I, three cable grafts were connected from the single ruptured root to the anterior division of the upper trunk for elbow flexion reconstruction. In group II, elbow flexion reconstruction was done by a distal nerve transfer either from the ulnar or median nerve. This provided more cable grafts for reconstruction of the posterior division of the upper trunk and the middle trunk in group II when compared to group I as shown in Table 3.

**Table 2:** Distribution of root rupture and avulsion in the two study groups

Root	Group I (*n* = 29)	Group II (*n* = 26)
C5	26 ruptured, 3 avulsed	24 ruptured, 2 avulsed
C6	2 ruptured, 27 avulsed	1 ruptured, 25 avulsed
C7	1 ruptured, 28 avulsed	1 ruptured, 25 avulsed

**Table 3:** The method of primary reconstruction of the brachial plexus in the two study groups

Motor function to be reconstructed	Group II	Group II
C5 root function		
- Shoulder abduction (the posterior division of the upper trunk) - Shoulder external rotation (suprascapular nerve)	One cable graft from the ruptured root to the posterior division of the upper trunk Spinal accessory to suprascapular nerve transfer	Two or three cable grafts from the ruptured root to the posterior division of the upper trunk Spinal accessory to suprascapular nerve transfer
C6 root function		
- Elbow flexion (the anterior divison of the upper trunk)	Three cable grafts from the ruptured root to the anterior division of the upper trunk	Distal nerve transfer (one fascicle from the ulnar or median nerve to the biceps nerve)
C7 root function		
- Neurotization of the middle trunk for elbow/wrist/digital extension	One cable graft from the ruptured root to the middle trunk	Two or three cable grafts from the ruptured root to the middle trunk

Table 4 shows the results for should abduction at 3 years. Shoulder abduction ranged from 30° to 100° (mean of 60°) in group I; and the percentage of a satisfactory outcome was 0%. In contrast, shoulder abduction ranged from 50° to 180° (mean 109°) in group II, and 42.3% of patients qualified for a satisfactory outcome. The outcome was significantly better in group II (*P* < 0.0001 by the Chi-Square test).

**Table 4:** Shoulder abduction at 3 years in the two study groups

Active shoulder abduction in degrees	Group I (*n* = 29)	Group II (*n* = 26)
Less than 30°	0	0
30°	2	0
40°	6	0
50°	5	2
60°	7	1
70°	2	3
80°	3	1
90°	2	4
100°	2	2
110°	0	2
120°	0	2
130°	0	3
140°	0	1
150°	0	1
160°	0	0
179°	0	3
180°	0	1

For external rotation of the shoulder (Table 5), the percentage of a satisfactory outcome (a score of 3 or more) was higher in group II (57.7%) than that in group I (48.3%); but the difference did not reach statistical significance (*P* = 0.485 by the Chi-Square test).

**Table 5:** Shoulder external rotation at 3 years in the two study groups

Shoulder external rotation score^a^	Group I (*n* = 29)	Group II (*n* = 26)
1	7	3
2	8	8
3	7	4
4	7	11
5	0	0

^a^The score as per Table 1

Except for one patient in each group, all patients in both groups obtained a satisfactory elbow flexion score (Table 6). A score of 4 or 5 was obtained in 96.6% of group I patients and in 96.2% of group II patients; and the difference between the two groups was not significant (*P* = 0.727 by Fisher’s exact test). Similarly, all patients in both groups qualified for a score of 4 of elbow extension (100% satisfactory outcome). The reason behind not having full elbow extension was a mild elbow flexion contracture (10°–30°) which was noted in all patients in both groups. Forearm pronation/supination was also satisfactory in all patients in both groups (Table 7). No statistical testing was done for elbow extension or forearm rotation because the outcome was satisfactory in all patients in both groups for these two functions.

**Table 6:** Elbow flexion at 3 years in the two study groups

Elbow flexion score^a^	Group I (*n* = 29)	Group II (*n* = 26)
0	0	1
1	0	0
2	0	0
3	1	0
4	4	2
5	24	23

^a^The score as per Table 1

**Table 7:** Forearm pronation/supination at 3 years in the two study groups

Forearm pronation/supination score^a^	Group II (*n* = 29)	Group II (*n* = 26)
1	0	0
2	0	0
3	23	22
4	6	4
5	0	0

^a^The score as per Table 1

Wrist extension scores are shown in Table 8. Nine patients (31.0%) in group I and 20 patients (76.9%) in group II obtained a satisfactory outcome and the difference was significant (*P* = 0.001 by the Chi-Square test). Digital extension scores are shown in Table 9. Twenty-five patients (86.2%) in group I and 25 patients (96.2%) in group II obtained a satisfactory outcome and the difference between the two groups was not significant (*P* = 0.212 by Fisher’s exact test).

**Table 8:** Wrist extension at 3 years in the two study groups

Wrist extension score^a^	Group I (*n* = 29)	Group II (*n* = 26)
0	5	3
1	15	3
2	9	15
3	0	5

^a^The score as per Table 1

**Table 9:** Digital extension at 3 years in the two study groups

Digital extension score^a^	Group I (*n* = 29)	Group II (*n* = 26)
0	0	0
1	4	1
2	10	6
3	15	19

^a^The score as per Table 1

## Discussion

The use of the spinal accessory to suprascapular nerve transfer is a well-established procedure for several decades [13–15]; and is usually done in brachial plexus injuries with root avulsion. When there is no root avulsion, the suprascapular nerve is usually neurotized using the C5 root. There are two studies in the literature comparing suprascapular nerve reconstruction in OBBP with either nerve grafting from C5 or the spinal accessory nerve transfer [16, 17]. The outcome of both methods was similar in both studies [16, 17]. In the current study, the spinal accessory transfer was used in all patients because of the presence of root avulsion. The outcome of external rotation was similar in both groups: about 50% could reach the ear or the back of the head. It is important to note that children with this satisfactory functional outcome utilize the help of shoulder abduction to reach the head. Hence, this satisfactory outcome is not a measure of true external rotation of the shoulder as demonstrated by Pondaag et al. [17]. It is also important to note that we do not use the Mallet score to assess the shoulder in OBBP because we noted that several children will have a discrepancy between the deficiency in shoulder abduction and shoulder external rotation. Hence, we document the outcome of these two shoulder functions separately as recommended by Al-Qattan and El-Sayed [18].

In adult traumatic brachial plexus injuries, isolated C5 and C6 avulsion is a common injury. In these cases, elbow flexion reconstruction is usually done using distal nerve transfers utilizing a single fascicle from the ulnar nerve to the biceps nerve (which was first reported in adults by Oberlin et al. in 1994) [19], a single fascicle from the median nerve to the biceps nerve, or double fascicular transfer (one fascicle from the ulnar nerve and another from the median nerve) to re-innervate the biceps and brachialis muscles [20]. In OBBP, the first use of distal nerve transfer for biceps reconstruction was in 2002 [11] and this was followed by several series utilizing the ulnar nerve, median nerve, or both in the double fascicular transfer [21–26]. The results in infants have been excellent, and this has been observed in the current series.

In adults, Oberlin nerve transfer gives better results in cases of C5-C6 avulsion when compared to those with C5-C6-C7 avulsion [27, 28]. Our study shows that biceps distal nerve transfer in OBBP gives excellent results even in the presence of C7 avulsion; which was present in the majority of cases in both groups (Table 2).

The most unique feature of the current study is the comparison between two reconstructive strategies in infants with extended Erb’s palsy and double root avulsion. Both groups were operated upon by the same surgeon and both had similar operative findings. In the first group, elbow flexion reconstruction was done by nerve grafting from the available ruptured root to the anterior division of the upper trunk. In cases of extended Erb’s palsy with double root avulsion, it is expected that the surgeon will give the priority to elbow flexion and not to shoulder abduction or wrist extension. Hence, most of the cable grafts were utilized for elbow flexion at the expense of shoulder abduction and wrist extension. In the second group, the use of a distal nerve transfer for elbow reconstruction provided more cable grafts for shoulder abduction and wrist extension reconstruction. Hence, it is of no surprise that the outcome of shoulder abduction and wrist extension was significantly better in the second group.

The excellent recovery of elbow extension and digital extension in both groups is probably attributed to the fact that the triceps and digital extensors are supplied by both the C7 and C8 roots; and the latter root is intact in both study groups.

All patients in both groups had avulsion of two roots and rupture of the third root. Hence, a single root was used for neurotization. The average number of nerve fibers in the C5 root (16,472 fibers) is much lower than the number in the C6 (27,421fibers) and the C7 (23,781 fibers) roots [2]. In our series, the root used for neurotization was the C5 root in the majority of cases in both groups (see Table 2). Therefore, the available number of fibers was similar in the two study groups.

Theoretically, group II had an advantage over group I with regard to the risk of postoperative co-contraction of elbow flexors and extensors. In group II, the biceps was neurotized separately by a distal nerve transfer; and hence, there was no risk of co-contraction. In contrast, group I had neurotization of both elbow flexors and extensors from the same root, hence the risk of co-contraction. We did not specifically assess co-contraction in our series; but a satisfactory elbow flexion was seen in 28 out of 29 patients in group I indicating the co-contraction was not a major concern.

In conclusion, the use of distal nerve transfer for biceps reconstruction in extended Erb’s obstetric palsy with double root avulsion gives a better outcome for shoulder abduction and wrist extension; and this seems to be related to the availability of more cable grafts to the posterior division of the upper trunk and to the middle trunk.
